# Outcomes of a culturally informed weight-loss competition for New Zealand Indigenous and Pacific peoples: a quasi-experimental trial

**DOI:** 10.1186/s40795-021-00457-9

**Published:** 2021-09-10

**Authors:** Marewa Glover, Anette Kira, Hayden McRobbie, Rozanne Kruger, Mafi Funaki-Tahifote, Jane Stephen, Bernhard H. Breier, Geoff Kira

**Affiliations:** 1 School of Health Sciences, College of Health, Massey University, Auckland, New Zealand; 2 Manawatū, New Zealand; 3 Lakes District Health Board, New Zealand and National Drug and Alcohol Research Centre, UNSW, Sydney, Australia; 4 School of Sport, Exercise and Nutrition, College of Health, Massey University, Auckland, New Zealand; 5 Pacific Heartbeat, Heart Foundation, Auckland, New Zealand

**Keywords:** Obesity prevention, Weight-loss competition, Indigenous, Culturally-based interventions

## Abstract

**Background:**

Reducing obesity prevalence among marginalised subgroups with disproportionately high obesity rates is challenging. Given the promise of incentives and group-based programmes we trialled a culturally tailored, team-based weight-loss competition with New Zealand Māori (Indigenous) and Pacific Island people.

**Methods:**

A quasi-experimental 12-months trial was designed. The intervention consisted of three six-months competitions, each with seven teams of seven members. Eligible participants were aged 16 years and older, with a BMI ≥30 kg/m^2^ and being at risk of or already diagnosed with type-2 diabetes or cardiovascular disease. Height, weight and waist circumference were measured at baseline, 6 and 12 months.

**Results:**

Recruitment of a control group (*n* = 29) versus the intervention (*n* = 132) was poor and retention rates were low (52 and 27% of intervention participants were followed-up at six and 12 months, respectively). Thus, analysis of the primary outcome of individual percentage weight loss was restricted to the 6-months follow-up data. Although not significant, the intervention group appeared to lose more weight than the control group, in both the intention to treat and complete-case analyses.

**Conclusions:**

The intervention promoted some behaviour change in eating behaviours, and a resulting trend toward a reduction in waist circumference.

**Trial registration:**

ACTRN12617000871347 Registered 15/6/2017 Retrospectively registered.

**Supplementary Information:**

The online version contains supplementary material available at 10.1186/s40795-021-00457-9.

## Background

People with a body mass index (BMI) greater than 30 have a higher risk of a range of illnesses, such as heart disease and type 2 diabetes mellitus (T2DM) [1]. In high income countries with an Indigenous population who, as a result of colonisation, have been marginalised – such as in the United States, Canada, Australia and New Zealand (NZ) – the Indigenous people have a higher prevalence of obesity than the dominant European population [2]. In 2019, Māori and NZ-resident Pacific Island adults were over-represented among people with obesity compared to their counterpart groups (Māori versus non-Māori by 1.8 times; and Pacific versus non-Pacific by 2.5 times) [3].

A range of interventions, from policy to individual treatment, are required to tackle this disparity. Interventions that are aimed at helping individuals from Māori and Pacific populations to lose weight are urgently needed. It has been proposed that health interventions for Indigenous peoples could be more attractive and effective if they are based on Indigenous theoretical frameworks [4]. As early as 1989, research indicated that a weight-loss competition may be a suitable strategy for Indigenous people [5]. There has been a call to adopt a group-focused approach to weight loss for Indigenous people in America, so as to: “(a) build and reinforce social cohesion and collective efficacy, (b) use the motivating force of friendly competition, and (c) aspire to change local norms and policies through assuring high visibility of alternate behaviors and engaging formal and informal leaders” [6 , p 224]. Group weight-loss competitions, including social media elements, have grown in popularity in recent years [7–9] with some finding efficacy for some participants [10].

Like Native Americans [6], Māori and Pacific people are tribal groups with a history of friendly inter-tribal rivalry and competition [11–13]. Māori and Pacific people have high participation rates in organised competitions and events [14], such as regional and national cultural performing arts competitions [15] and traditional sports such as kilikiti (a Samoan form of cricket), which incorporate competitiveness with cultural elements [16, 17].

One successful culturally-based intervention in NZ was a team quit-smoking competition called WERO [11]. WERO (Whānau [family] End smoking Regional whānau Ora [health] challenge) combined a number of culturally salient components: commitment-to-the-group and between-group competition and financial incentives, which were donated to a nominated charity. The competition was backed up with pharmacological treatments for cessation, cognitive behavioural treatment delivered via an interactive website and locally-based health providers. The WERO intervention achieved a high biochemically verified quit rate of 36% at three months, which represented the potential to substantially reduce smoking for an important NZ population group.

Although the WERO intervention was successful for helping Māori and Pacific people to stop smoking, it had not been tested for changing other health-related behaviours, such as weight loss. Therefore, the current research aimed to test if a culturally- and community-based team intervention (called Reducing Weight through Eating Healthy and Increasing activity (WEHI) modelled on the WERO intervention), might be effective in helping NZ Māori and Pacific people to lose weight by improving healthy eating behaviours and increasing physical activity of teams. This paper reports on the main outcome of weight loss.

## Methods

A quasi-experimental trial comparing weight-related anthropometric changes following a six months intervention against a control group receiving no intervention, with a follow-up at 12 months was planned. The rationale and method for the WEHI trial are described in detail elsewhere [18]. This section briefly describes the methodology.

### Participants and sample size

Three distinctly different geographical regions in NZ were selected for recruitment: an urban Māori population (Palmerston North), a small town/rural Māori population (Northland) and a Pacific Island community in a major NZ city (Auckland). Seven teams of seven members for each region (total *N* = 147) were recruited for the intervention. We planned to recruit 150 wait list control participants, who did not receive any intervention (see below).

### Eligibility and exclusion criteria

The eligibility criteria were Māori or Pacific people, aged 16 years of age and above, having a body mass index (BMI) ≥ 30 kg/m^2^, and being at risk of or having developed T2DM or cardiovascular disease (CVD). People who were of any other ethnicity, younger than 16 years, pregnant or were breastfeeding and had type 1 diabetes were excluded. To control for potentially confounding factors, using nicotine from any source (a known appetite suppressant [19]) or smoking cannabis (with debated effects on weight [20]) were additional exclusion parameters.

### Recruitment

The intervention group was purposively recruited by Māori and Pacific health providers. They used convenience sampling to find participants, advertising through their existing networks to staff and in their communities. Though recruitment of intervention and control participants occurred concurrently, recruitment for control participants was done over an extended time period (four months). Potential participants who declined the intervention were invited to enrol as a control participant, or they responded directly to invites to be a control participant. In lieu of receiving the intervention, control participants were offered one entry in a prize draw (one per region) for petrol vouchers (up to $50) for completing a questionnaire at baseline, six-month follow-up and 12-month follow-up.

### Intervention

The WEHI intervention included four main components: group support, competition, financial incentives and Internet-delivered education and support. Teams were to meet regularly to facilitate their completion of as many of the competition’s weekly and nine daily challenges as they could to amass points. The activities were designed to prompt physical activity, increase consumption of vegetables, reduce consumption of added-sugar drinks and foods, and encourage retention in the competition (see Glover et al. [21] for a fuller description and evaluation). Each team had seven members and were self-directing, however, they could receive support from regional intervention workers. Support was also provided via the intervention website which provided weight-loss tips and answers to questions on, for example, staying motivated, making choices and increasing physical activity. Each team also had a dedicated team page where they could post photos, recipes and comments. This was also publicly visible as was a competition scoreboard displaying the progress of each team. In each region, three cash prizes were offered for: the greatest progress at two months (NZ$1000), greatest progress at four months (NZ$1000) and greatest progress at six months (NZ$3000). Progress was based on the number of team members who had lost ≥4 kg weight in the preceding two months plus the number of team members who had lost ≥3 cm in waist circumference during the same period, plus the team’s position on the competition scoreboard, which was calculated by tracking team participation and completion of daily and weekly challenges. The prizes were paid to the team’s nominated charity or community organisation.

### Measures

Anthropometric measurements (height, weight and waist circumference) of all participants were performed by the researchers or research assistants. The equipment used for measurements were a SECA813 digital floor scale [22], a SECA portable stadiometer height rod [23] and a SECA ergonomic girth measuring tape [24]. Based on those measures BMI was calculated as (weight in kilograms (kg) / (height meter (m)2)).

Questionnaires (see supplementary file) were self-administered at baseline, 6 months and 12 months to measure changes in Ministry of Health Eating and Activity Guidelines [25] eating goals, such as eating two servings of fruit a day, activity levels, perceived acceptability of WEHI. Other questions at baseline asked about previous use of dieting/weight-loss programmes, demographic characteristics and food security. In addition to food security, being a holder of a community services card was used as a proxy indicator of socioeconomic status. Community services cards enable people on low incomes to receive discounts, for instance on their healthcare and cost of medications. The questions were drawn or adapted from other surveys, such as the Adult Nutrition Survey, Ministry of Health adult health survey, food security index and relevant literature. The questionnaire was pilot tested with six Māori and Pacific people with BMI > 30 from among the researchers’ networks.

### Outcomes

The primary outcome was mean weight loss at six-month follow-up. Secondary outcomes included the proportion of participants losing at least 5 and 10% of baseline weight, change in waist circumference and BMI at six-month follow-up. We also looked at weight-loss outcomes in the intervention group at 12-month follow-up.

### Data analysis

A simple descriptive analysis, calculating counts and percentages, was performed on ordinal data variables. Baseline weight, waist circumference and BMI were not normally distributed and so non-parametric tests were used to examine differences between groups. Where indicated, additional analyses were calculated for subcategories. For continuous variables, mean, standard deviation and maximum and minimum values were calculated. Differences between groups were compared using an univariate general linear model, adjusting for baseline body weight. To assess differences in the proportion of baseline body weight lost, a chi-square test was used. An intention-to-treat (ITT) analysis was used. We used a baseline-observation-carried-forward-analysis for those who were lost to follow-up. We also undertook a complete-case analysis. To calculate change in eating behaviour a non-parametric Related-Samples Wilcoxon Signed Rank Test was performed to detect if there was a significant difference (*p* < 0.05) between eating behaviour at baseline versus six months. All analyses were undertaken using IBM SPSS.

### Ethics

This study was approved by the NZ Ministry of Health’s Northern B Health and Disability Ethics Committee (16/NTB/101).

## Results

A total of 181 people expressed an interest in joining the study, but 12 decided to not participate leaving 140 in the intervention group and 29 in the control group. Participants in the intervention group were divided into 20 groups (18 groups of 7, 1 of 6 and 1 of 8). Six participants withdrew from the study and two never started, leaving 132 and 29 participants in the intervention and control groups, respectively. The demographics and baseline anthropomorphic data are given in Table 1.

**Table 1:** Characteristics of participants in the WEHI study

Measure	Intervention *N* = 129–132^a^ (%)	Control *N* = 28–29 (%)	Difference
Female	106 (82.2%)	23 (82.1%)	NS
Māori	77 (58.3%)	19 (67.9%)	NS
Pacific	52 (39.4%)	8 (28.6%)	
European/Other	3 (2.3%)	1 (3.6%)	
Have a community services card?			chi-square = 6.8, *p* = 0.078
Yes	39 (30.2%)	4 (14.3%)	
No	67 (51.9%)	22 (78.6%)	
Don’t know	23 (17.9%)	2 (7.1%)	
Baseline weight (kg)			*p* = 0.022
mean (SD)	114.0 (21.3)	103.4 (16.9)	
Min	73.5	75.5	
Max	176.4	130.0	
Baseline waist circumference (cm)			*p* = 0.013
Mean (SD)	118.4 (14.4)	111.0 (11.5)	
Min	92.2	91.4	
Max	164.0	137.5	
Baseline BMI (kg/m^2^)			p = < 0.001
Mean (SD)	41.1 (6.4)	36.4 (4.9)	
Min	30.3	30.2	
Max	59.3	49.1	
Follow-up attendance (n (%))			
2 months N (%)	93 (70.5%)	–	
4 months N (%)	61 (46.2%)	–	
6 months N (%)	69 (52.3%)	16 (55.2%)	
12 months N (%)	36 (27.3%)	–	

^a^Ns vary due to missing data

Retention rates were low, with only 52 and 27% of participants recruited into the intervention group being available for follow-up at six and 12 months, respectively. The study struggled to recruit participants for the control group, and follow-up of these participants was difficult. An initial 29 control participants were recruited and of those 55% (*N* = 16) were still in the study at six months. Due to the high proportion lost to follow-up at six months and a small sample, 12-month follow-up of the control group was not conducted. Too few of the intervention group remained at the 12-month follow-up to conduct analysis of within group weight-loss difference. Thus, analysis of weight-loss is restricted to the 6-month follow-up data.

### Weight loss at 6 months of intervention

The intervention group lost more weight than the control group, in both the ITT and complete-case analyses and the average weight loss from baseline was statistically significant in the intervention group. However, the differences between groups were not statistically significant (see Table 2). A slightly higher proportion of participants in the intervention group lost at least 5% baseline body weight, but these differences were not statistically significant (see Table 3).

**Table 2:** Change in anthropomorphic outcomes at six months

	Intervention	Control	Difference^a^
Intention-to-treat	N = 132 mean (95% CI)	N = 29 mean (95% CI)	
Change in weight (kg)	−2.1 (−3.0 to − 1.2)	− 1.6 (− 3.6 to 0.3)	F = 1.1, *p* = 0.3
Complete case	*N* = 69 mean (95% CI)	N = 16 mean (95% CI)	
Change in weight (kg)	−4.1 (−5.7 to − 2.4)	−2.5 (− 5.9 to 0.9)	F = 0.7, *p* = 0.4
Change in waist circumference (cm)	−6.8 (−8.9 to − 4.7)	− 2.8 (− 5.4 to − 0.2)	F = 3.2, *p* = 0.08
Change in BMI	−1.5 (− 2.1 to − 0.8)	−0.9 (− 1.9 to 0.2)	F = 0.8, p = 0.4
% of baseline body weight lost	3.6% (2.1 to 5.1%)	2.2% (− 0.7 to 5.1%)	F = 0.7, p = 0.4

^a^Adjusted for baseline weight

**Table 3:** Proportion losing 5 and 10% of baseline body weight at six-month follow-up

	Intervention N = 132 (%)	Control *N* = 29 (%)	Difference
lost at least 5% of baseline body weight	21 (15.9%)	4 (13.8%)	chi-square = 0.08, *p* = 0.8
lost at least 10% of baseline body weight	9 (6.8%)	1 (3.4%)	chi-square = 0.5, *p* = 0.5

### Eating behaviour

Changes in eating behaviours were not assessed in the control group due to missing data and small sample size. Table 4 summarises the change in selected eating behaviours that the intervention was designed to change among the intervention participants. The average is shown for the whole group, for whom data existed, at each data collection point. Changes were more likely to be detected at the end of the competition (at six months) as opposed to at 12 months (data not shown) follow-up. Significant changes between intervention group behaviour at baseline and at six months were found for several behaviours the Ministry of Health Eating and Activity Guidelines for New Zealand Adults (25) provides advice on. These included: increased servings of fruit per day consumed, increased days per week consuming vegetables, increased times eating low fat meals per week and increased quantity of unflavoured water consumed per day. Reductions were seen in portions of fat used on bread or added to meals, frequency of consuming sugar-sweetened drinks and eating sweets, and fast foods per week.

**Table 4:** Eating behaviour changes from baseline to six months in the intervention group

Competition intervention group behaviour objectives	Baseline ± Standard Deviation (n)	6 Months ± Standard Deviation (n)	Median difference Related-Samples Wilcoxon Signed Rank Test *p* Value
fruit servings per day	1.8 ± 1.0 (*n* = 127)	2.6 ± 1.7 (*n* = 62)	0.000 * (*n* = 61)
days eating vegetables per week	4.9 ± 1.8 (*n* = 119)	5.9 ± 1.5 (*n* = 60)	0.005* (*n* = 55)
vegetable servings per day	2.3 ± 1.4 (*n* = 124)	2.9 ± 1.9 (n = 60)	0.166 (*n* = 56)
butter/margarine/meat fat portion size	2.5 ± 1.2 (*n* = 122)	1.7 ± 1.1 (*n* = 57)	0.000* (n = 55)
times eating low fat meals in last week	2.6 ± 1.0 (n = 122)	2.0 ± 0.9 (n = 57)	0.000* (*n* = 54)
times eating fast food in last week	2.8 ± 1.6 (*n* = 125)	1.8 ± 1.5 (n = 61)	0.000* (*n* = 59)
times drank sugar-added drinks	2.5 ± 1.9 (n = 124)	1.2 ± 1.4 (n = 61)	0.000* (n = 60)
unflavoured water (litres) per usual day	1.3 ± 1.0 (*n* = 128)	1.8 ± 1.0 (n = 62)	0.005* (n = 61)
times eating sweets in last week	3.0 ± 1.8 (n = 125)	2.1 ± 2.0 (n = 60)	0.001* (*n* = 58)
times eating breakfast in last week	4.6 ± 2.3 (*n* = 126)	4.9 ± 2.4 (n = 60)	0.855 (n = 59)

*statistically significant at *p*<0.05

## Discussion

The WEHI intervention appeared to promote change in eating behaviour but these changes did not result in any significant change in body weight at six months, compared to participants (controls) who received no intervention. There were trends toward a reduction in waist circumference for the Māori and Pacific groups in this study.

Two major limitations of this study were the small size of the control group and high rates of attrition. Both of these factors limited the power of the analyses to evaluate the effect of the WEHI trial. The lack of intervention for control participants was a disincentive to enrol limiting participation in the study. Further, the control participants had, on average, a lower BMI and proportionately higher educational levels (found to attenuate weight loss programme attrition [26]). This limited the robustness of comparisons that could be made between the intervention and control participants.

The current trial suffered from average attrition which is not uncommon for weight-loss interventions with the rate of attrition having been found to vary as much as 10–80% [26]. Furthermore, recruiting and retaining Indigenous people in controlled trials is known to be difficult [27]. Despite following recommendations from the literature (see Glover et al. [18]), recruitment of control participants and retention for both intervention and control participants were a problem for this study. One potential reason for the attrition was that two providers recruited internally to their organisations. Therefore, staff may have felt compelled to participate, but were not actually motivated to lose weight or to participate in the intervention. Unfortunately, motivation to lose weight was not measured at screening or baseline. The timing of the WEHI competition which ran through the Christmas period when most staff of community health organisations take extended holiday leave was another cause of intervention participant drop-out [21].

It was anticipated that the financial incentives provided in this study would support intervention group retention. It emerged that most participants in the five teams who won or shared in a progress prize were sufficiently motivated to persist to the end of the six-month intervention. The receipt of prizes throughout the competition may have contributed to team members’ motivation to maintain behaviours. Interestingly, the five teams who won some prize money continued competing after winning. Not winning may have conversely acted to demotivate continued participation among those teams who had members drop-out. Chin et al. [28] found that incentivising attendance versus only rewarding weight loss was associated with less attrition and more weight loss, though intervention dose was important regardless of the incentive strategy. Despite offering incentives they concluded that enrolment and retention remained challenging.

Research has indicated that financial incentives are effective for assisting people to attend and persist with weight-loss interventions resulting in weight loss [28–30]. However, despite including financial incentives in WEHI, the desired weight loss was not achieved. It is possible that the way and frequency in which the incentives were delivered needs to be revised. Burns et al. [30] in their systematic review suggest that an incentive that is contingent on an outcome and is continuous throughout the intervention is more effective than a lottery type reward. Restructuring the incentives and increasing the reward might make similar interventions more effective. However, a more recent review of the use of incentives to change health behaviours concluded that efficacy is highly context-dependent and will vary across demographic groups and target behaviours [31].

A strength of this study was the design of the WEHI intervention, by and for Indigenous people, which made it attractive enough to enrol participants who have been perceived to be difficult to recruit, that is, both as people with obesity and Indigenous. Another strength is that WEHI participants were recruited from the general population, versus a clinical population advised to seek treatment for obesity. This showed that the WEHI programme is pragmatic enough to be delivered within the existing health system. A final strength is enlisting community providers to conduct some of the research tasks. This builds both community understanding of research and local capability.

The WEHI trial showed promise for health behaviour change, which is important for long-term weight loss. The intervention appeared to be effective in changing some dietary behaviours in the short term. Other authors have suggested that small changes, especially if triggered by low-cost interventions that remotely facilitate self-monitored or self-monitored with tailored feedback, could produce a significant public health impact if extrapolated over a population [32].

The WEHI trial also contributes valuable information in an area that has been substantially under-researched: weight-loss programmes designed by and for Indigenous people.

### Future work

While competitions are attractive for some people, they are not likely to be attractive to others. Future trials may need longer recruitment times and more training and support for community partners in order to improve recruitment rates. Where an intervention is highly attractive, a wait-list control design could aid in the enrolment and retention of a control group.

More research is needed on which combination of technological and incentive intervention components will increase efficacy. WEHI incentives were based on group and individual progress. Interventions could retain competition, which has been related to weight loss changes [33], but refrain from publicly displaying how participants or teams are progressing so as not to disincentivise teams who see their chance of winning reducing as others progress at a faster pace. Future research is needed to identify optimal use of incentives for triggering behavioural change and adherence to weight-loss goals, such as increasing the relative reward for higher impact behavioural change objectives and reduced consumption of fast foods over the reward for less impactful goals, such as drinking more water. Potential alternatives to test include: awarding a proportion of the prize relative to individual or team effort, for example a nominal amount per percentage weight lost; increasing the frequency of incentives to support continuous motivation; and payment of an incentive to individual participants, as opposed to donating the award to a charity or community organisation. This will allow a larger breadth of participants to be able to receive incentives.

Similar future trials should incorporate qualitative evaluation tasks to enable analysis of contextual factors that may impact the intervention. We did analyse adherence [21] utilising programme data, and we did conduct some qualitative work assessing the acceptability of the intervention to a sub-sample of participants’ and regional co-ordinators’, but that work remains unpublished (those results are contained in a technical report prepared for the funder which is available from the author).

Group weight-loss competitions have predominantly been researched in workplaces. The involvement of work-based teams in WEHI suggests that there could be merit in trialling a version of WEHI specifically focused on Māori and Pacific health workplaces. One of the benefits of WEHI being delivered into a workplace is that social practices that encourage over-eating and or consumption of foods that undermine weight-loss goals can be highlighted for change.

## Conclusion

Given the dearth of previous weight-loss programmes available in many Māori communities, and the higher proportion of Māori and Pacific people with obesity, this study provides initial information useful for designing acceptable and attractive interventions for these high priority groups. The WEHI trial was successful at triggering some Government recommended dietary changes, such as eating a minimum of two servings of fruit a day and some effect on weight loss was indicated. Even though the effect was small, programmes like WEHI that can cost-effectively reach a large number of people could deliver significant public health gains if delivered at a population level. Like the WERO stop smoking competition, WEHI could be delivered as a health promotion programme with the aim of triggering weight-loss attempts, rather than as a personal health treatment programme. Its attractiveness and efficacy in supporting people from dispersed, rural and high priority groups, and feasibility for use in workplaces suggests the concept is worth further refinement and testing. Obviously, there is a need to improve retention of participants in the intervention, but this is a global challenge for weight-loss programmes.

## Supplementary Information

**Additional file 1.**

## Data Availability

Participant consent did not include consent for posting the data in an online repository. The datasets used and/or analysed during the current study are available from the corresponding author on reasonable request.

## Abbreviations

BMI: body mass index
CVD: cardiovascular disease
NZ: New Zealand
SD: standard deviation
T2DM: type 2 diabetes
