# Supplementary material for: Outcomes of a culturally informed weight-loss competition for New Zealand Indigenous and Pacific peoples: a quasi-experimental trial
Source: BMC Nutr. 2021 Sep 10;7:52. doi: 10.1186/s40795-021-00457-9 (PMC8431855; doi:10.1186/s40795-021-00457-9)
Supplement: Supplementary file 1 — Additional file 1. [file 40795_2021_457_MOESM1_ESM.pdf]

ASK THESE QUESTIONS TO SEE IF PEOPLE ARE ELIGIBLE FOR WEHI

Q1 In which month and year were you born?	Month ______________Year _______________	***Aged less than 16+ = not eligible***

If female aged 16-55yrs ask:<br>Q2 Are you currently pregnant, breastfeeding or planning on becoming pregnant in the next 12 months?	○ Not applicable (male, not of child-bearing age, *skip to Q3*)<br>○ Yes = ***they are not eligible. End questioning*.**<br>○ No
Q3 Do you smoke tobacco or take nicotine (e.g. from gum, vaping, lozenge) regularly at least once a month?	○ Yes = ***they are not eligible. End questioning*.**<br>○ No

Below 30 BMI = Not eligible. Take the following physical measurements 2 times. If measure 1 & 2 are very different, take a 3rd. Then use final average amounts to calculate BMI (weight ÷ (height x height)) =

	**1st Measure**	**2nd Measure**	**3rd Measure**	**Average - Final**
Q4 Height (cm) (<0.5cm)	○	○	○	○
Q5 Weight (kg) (<0.1kg)	○	○	○	○
Q6 Waist circumference (cm) (<0.5 cm)	○	○	○	○

Q7 Have you ever been told by a doctor that you have diabetes?	○ Yes, pre-diabetes<br>○ Yes, Type I<br>○ Yes, Type II<br>O No<br>O Don’t know
Q8 What treatments do you <u>now</u> take for your diabetes? *(Tick all that apply)*	○ No treatment<br>○ Insulin injections<br>○ Medicines, tablets or pills<br>○ Other. Specify ____________________<br>○ Don’t know
Q9 Have you ever been told by a doctor that you have/had any of the following: *(Tick all that apply)*	○ Heart disease<br>○ High cholesterol<br>○ High blood pressure/hypertension<br>○ None of the above

If eligible, ask continue with *‘WEHI Questionnaire 1*

1 What region do you live in?	☐ Northland ☐ Auckland ☐ Manawatu
2 What are your initials?	
3 Are you:	☐ Male ☐ Female
4 Town where you live or nearest town:	
5 Do you have a mobile phone?<br>*(To find you for follow-up questionnaires)*	☐ Yes ☐ No<br><br>If Yes, what is your mobile phone number: ____
6 Do you have internet access?<br><br>*Tick all that apply.*	☐ No<br>☐ Yes, at home ☐ Yes, at work<br>☐ Yes, mobile ☐ Yes, somewhere else<br><br>If Yes, please provide your email address: ____
7 What is your **highest** completed qualification?<br><br>*Tick one.*	☐ None<br>☐ School Certificate OR National Certificate level 1 OR NCEA level 1<br>☐ Sixth Form Certificate OR National Certificate level 2 OR UE OR NCEA level 2<br>☐ Higher School Certificate OR Higher Leaving Certificate OR University Bursary OR National Certificate Level 3 OR NCEA level 3<br>☐ Bachelor's degree (e.g. BA, BSc)<br>☐ Diploma (not postgraduate)<br>☐ Master's degree (e.g. MA, MSc)<br>☐ PhD<br>☐ Trade or technical certificate (took more than 3 months study)<br>☐ Other. Specify ____
8 Which of these statements best describes your current work situation:	☐ Self-employed<br>☐ Working in paid employment<br>☐ Not in paid work, and looking for a job<br>☐ Not in paid work, and not looking for a job for any reason such as being retired, a homemaker, caregiver, or full-time student<br>☐ Other. Specify _______________________

9	Are you eligible for a Community Services Card?	☐ Yes ☐ No ☐ Don't know
10	Who lives in the same household as you?<br><br>*Tick as many spaces as you need to show all the people living with you.*	☐ Partner (husband / wife / boyfriend / girlfriend)<br>☐ Mother / mother in-law<br>☐ Sister / brother<br>☐ Flatmate or boarder<br>☐ Father / father in-law<br>☐ Children / Partners children<br><br>☐ Other. Specify ______________________________
11	What **medicines** are you on at the moment? You do not need to take this home, just do your best to remember and write the name of them. If you can't remember the name, what is the medicine for?	
	Name & Dose ______________________________<br><br>Name & Dose ______________________________<br><br>Name & Dose ______________________________<br><br>Name & Dose ______________________________	
12	What vitamins, supplements, laxatives or protein drinks or other diet pills have you used in the last month? You do not need to take this home, just do your best to remember and write the name of them. If you can't remember the name, what is the product for?	
	Name & Dose ______________________________<br><br>Name & Dose ______________________________<br><br>Name & Dose ______________________________	

13 How often do you have a drink containing **added** sugar, including but not limited to chocolate milk, milo, soft drink, sports drinks, energy drinks, tea and coffee?<br><br>*Don't include drinks with alcohol. Don't include 'diet' or 'zero' drinks that use artificial sweeteners instead of sugar.*	☐ Don't drink sugar sweetened drinks<br>☐ Once per week<br>☐ 2-3 times per week<br>☐ 4-5 times per week<br>☐ 1 a day<br>☐ 2-3 a day<br>☐ 4 or more a day
14 How much unflavoured water, including sparkling water, do you drink on a usual day?	☐ 0 – 250 mL<br>☐ 500-750 mL<br>☐ 1-1.5L<br>☐ 2 L or more<br>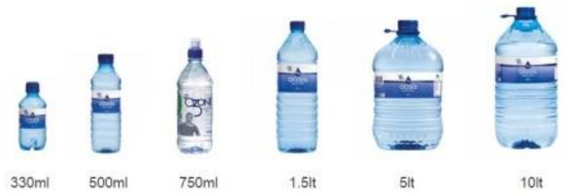
15 During the last 7 days, please circle, on how many days did you eat fruit?<br><br>*Please include all fresh, frozen, canned and stewed fruit. Do not include fruit juice or dried fruit.*	0-----1-----2-----3-----4-----5-----6-----7<br><br>☐ Don't know
16 On average, please circle, how many servings of fruit did you eat each day?<br><br>*A serving is 1 apple or 1 small banana or 2 mandarins or 2 kiwi fruit*	0-----1-----2-----3-----4-----5-----6-----7+
17 During the last 7 days, on how many days did you eat vegetables?	0-----1-----2-----3-----4-----5-----6-----7<br><br>☐ Don't know
18 On average, how many servings of vegetables did you eat each day?<br><br>*A serving is ½ - 1 cup carrots or salad or mixed vegetables*	0-----1-----2-----3-----4-----5-----6-----7+

19 How much margarine, butter or meat fat do you use to season vegetables or put on potatoes, bread or corn?	☐ 1 level teaspoon ☛ 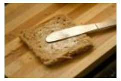<br>☐ 1 heaped teaspoon<br>☐ 1 tablespoon ☛ 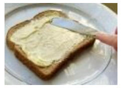<br>☐ 1 heaped tablespoon
20 How often do you choose low or reduced-fat varieties of food instead of the standard variety?	☐ Everyday<br>☐ 2-3 times per week<br>☐ 1-2 times per month<br>☐ Once per month or less
21 How often do you add salt to your food **after** it has been cooked or prepared?	0-----------1-------------2---------------3-------------4<br>Never Rarely Sometimes Often All the time

KA MAU TE WEHI awesome

During the last 7 days, how many times did you…	*Circle your answer:*
22 …eat fast food meals or snacks?<br>e.g. hot chips, pizza, sausage roll, burger or chicken nuggets	0-----1-----2-----3-----4-----5-----6-----7
23 eat regular snack chips or crackers?<br>e.g. ETA or Bluebird chips	0-----1-----2-----3-----4-----5-----6-----7
24 …eat desserts and other sweets?<br>e.g. cake, puddings, lollies, chocolate or biscuits	0-----1-----2-----3-----4-----5-----6-----7
25 …eat lean meat?<br>e.g. chicken thigh with no skin or unbattered fish or red meat with the fat trimmed	0-----1-----2-----3-----4-----5-----6-----7
26 …eat fresh seafood?<br>e.g. fish, mussels or pipi	0-----1-----2-----3-----4-----5-----6-----7
27 …eat legumes?<br>e.g. butter beans, chickpeas, hummus or lentils	0-----1-----2-----3-----4-----5-----6-----7
28 …have something to eat for breakfast?<br>e.g. porridge or cereal with milk, yogurt with fresh fruit, eggs on toast, a muffin	0-----1-----2-----3-----4-----5-----6-----7
29 What did you have for breakfast this morning?<br>*Write your answer here* ☛	

**30 If you were given a choice, which of the following foods would you choose? *(Tick one on each line)***		
2 chicken drumsticks	1 chicken breast	
2 sausages	1 lamb shank	
4 slices of bread	2 rolls	
½ cup pasta or rice	1 cup pasta or rice	2 cups pasta or rice
1 kumara	2 potatoes	1 cup mashed potato
1 muffin	2 slices of cake	4 Gingernut biscuits

**31 Tick the healthier way to cook from each pair:**	
Deep fry	Stir-fry
Boiled	Uncooked / raw
Steamed	Boiled
Shallow fried	Roasted
Grilled	Pan fried

32 Which cooking method do you use the most at home?	
33 Which of the above cooking methods do you think is the healthiest of all?	
34 Which cooking method do you think is the <u>least</u> healthy of all?	
35 An example of an energy-dense food is:<br><br>*Tick one*	☐ chocolate ice cream (290 calories per 1 cup)<br>☐ air-popped popcorn (30 calories per 1 cup)<br>☐ sliced fresh strawberries (50 calories per 1 cup)<br>☐ raw carrot sticks (50 calories per 1 cup)

36 Rank these foods, starting from 1 for the lowest number of calories. Calories are a measure of how much energy a food provides.

Write a number from 1 to 10 next to each food in this box:

____ Pint of beer 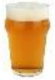

____ Large 150g pack potato chips 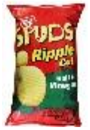

____ An apple 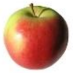

____ An ice cream cone 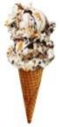

____ A pottle of yoghurt 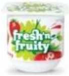

____ A glass of red wine 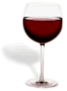

____ Hot chips 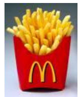

____ A small chocolate bar 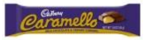

____ A cup of herb tea 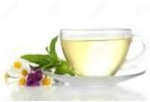

____ Flat white and muffin 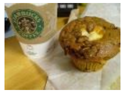

Write a number from 1 to 6 next to each food in this box:

____ Fish and chips 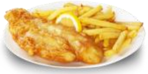

____ Chicken stir-fry with rice 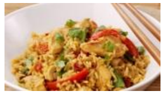

____ Tuna salad 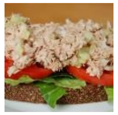

____ A Bic Mac & Fries 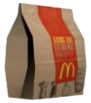

____ Beef stew with potatoes & vegetables 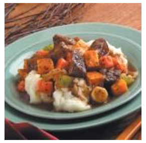

____ A tomato, ham and lettuce sandwich 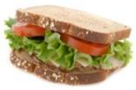

If you could eat one of these ☝ meals now, which one would you choose? *Tick one of the above pictures.*

**Thinking about the last 7 days:**	*Tick or Circle your answer:*
37 Who in your household usually decides what food and drink is bought?	☐ Me ☐ Me and others jointly<br>☐ Someone else
38 Who in your household usually decides how meals will be cooked?	☐ Me ☐ Me and others jointly<br>☐ Someone else
39 Do you usually dish up your own meals or are your meals plated up for you?	☐ I dish up my own meals<br>☐ Somebody else usually dishes up my plate
40 If you work, do people at work eat lunch together?	0-----------1-------------2---------------3-------------4<br>Never Rarely Sometimes Often All the time<br>☐ Not applicable
41 Food runs out in my/our household.	0-----------1-------------2---------------3-------------4<br>Never Rarely Sometimes Often All the time<br>☐ Don’t know
42 I/we make use of special food grants or food banks.	0-----------1-------------2---------------3-------------4<br>Never Rarely Sometimes Often All the time<br>☐ Don’t know
43 I feel stressed because of not having enough money for food.	0-----------1-------------2---------------3-------------4<br>Never Rarely Sometimes Often All the time<br>☐ Don’t know
44 The variety of food I am/we are able to eat is limited by a lack of money.	0-----------1-------------2---------------3-------------4<br>Never Rarely Sometimes Often All the time<br>☐ Don’t know

45 I/we rely on others to provide food and/or money for food for my/our household when I/we don't have enough money.	0-----------1--------------2----------------3--------------4<br>Never Rarely Sometimes Often All the time<br><br>☐ Don't know
46 How often do you have a drink containing alcohol?	☐ Don't drink any alcohol ☛ Go to Q. 49<br>☐ Never or rarely<br>☐ Less than monthly<br>☐ Once a month<br>☐ 2-3 times a month<br>☐ 1-2 times a week<br>☐ 3-4 times a week<br>☐ Once a day<br>☐ 2 times or more a day
47 How many drinks containing alcohol do you have on a typical day when you are drinking?	1-----2-----3-----4-----5-----6-----7-----8------9-----10-----11----12+<br><br>☐ Don't know
48 During the past four weeks, how much of the time have you done less than you would like as a result of any emotional problems, such as feeling depressed or anxious?	0--------------1-------------2--------------3--------------4<br>None of the time / A little the time / Some of the time / Most of the time / All of the time<br><br>☐ Don't know

**For questions 49 - 50 “activities” means doing anything using your muscles including activities you do at work, school, home, getting from place to place, for sport, recreation or leisure.**	
49 During the last 7 days, on how many days did you do **moderate** physical activity that made you breathe a little harder than normal?<br><br>*Think **only** about physical activities done for at least 10 minutes at a time.*	0-----1-----2-----3-----4-----5-----6-----7<br><br>☐ Can’t remember
49a What moderate physical activity did you do?	
49b How much time did you typically spend on each of those days doing moderate physical activity?	________ hours (range 0-24) _______ minutes (range 0-60)<br><br>☐ Don’t know
50 During the last 7 days, on how many days did you do **vigorous** physical activity that made you breathe a lot harder than normal (huff and puff)?<br><br>*Think **only** about physical activities done for at least 10mins.*	0-----1-----2-----3-----4-----5-----6-----7<br><br>☐ Can’t remember
50a What vigorous physical activity did you do?	
50b How much time did you typically spend on each of those days doing vigorous physical activity?	________ hours (range 0-24) _______ minutes (range 0-60)<br><br>☐ Don’t know
51 During the last 7 days, usually how many hours sleep each night did you get?	Less than 5---- 5-----6-----7-----8-----9-----10+<br><br>☐ Can’t remember

52 Looking at the pictures below, please circle the ONE which you feel is most similar in size to you.

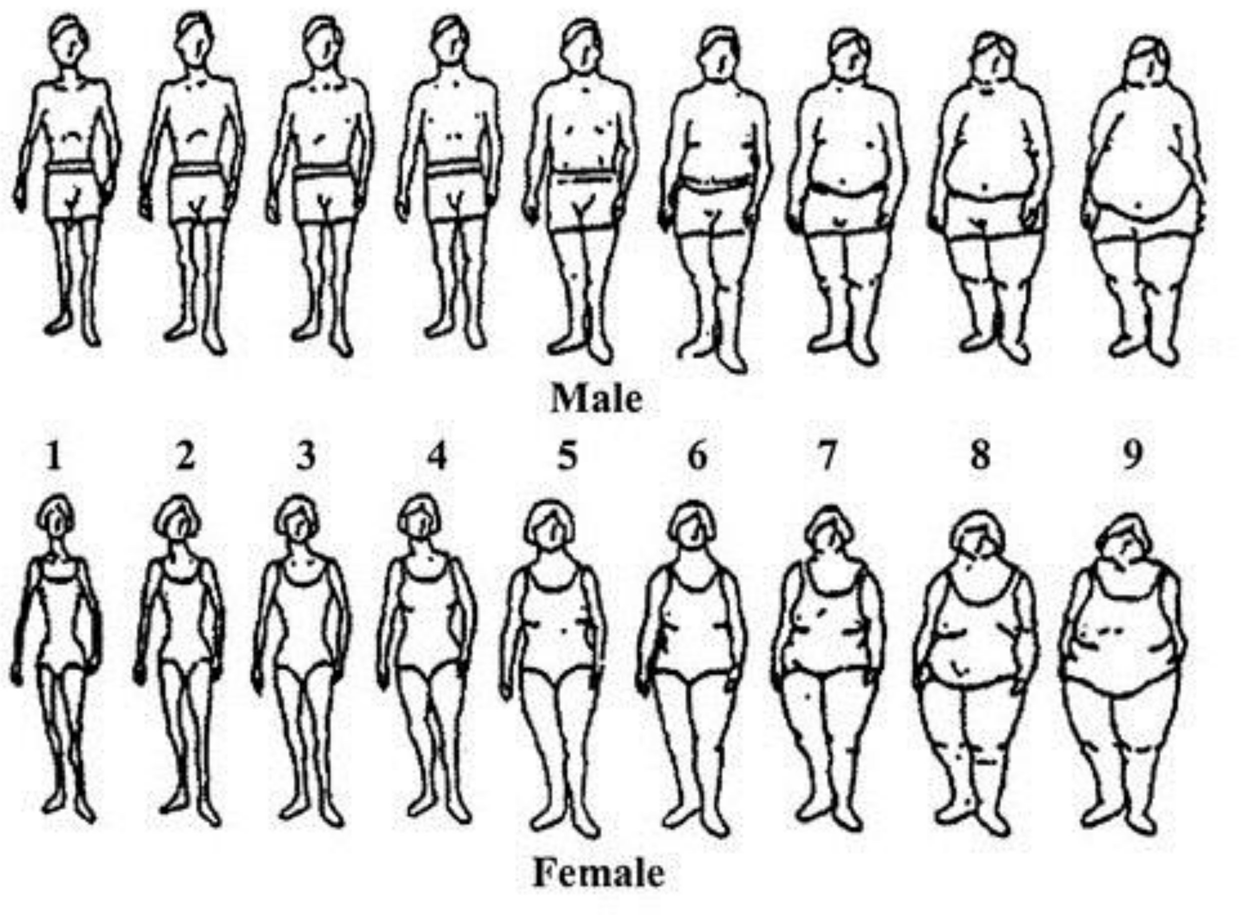

Q.53 Which, if any, of the pictures do you consider are:

(Write the numbers in the space provided)

____________ underweight

____________ neither under or over weight

____________ a little bit overweight

____________ a lot overweight

____________ seriously overweight

54 Are you **currently** on a weight loss programme or diet such as Weight Watchers, or a diet prescribed by a doctor or dietician?	☐ No<br><br>☐ Yes. Specify ________________________________
55 In the **past** 12 months, have you used any of the following diets or weight loss programmes to try to lose weight (excluding the one you may currently be following)?	☐ Weight Watchers<br>☐ Jenny Craig<br>☐ The Paleo Diet<br>☐ Ketogenic Diet<br>☐ Dash Diet<br>☐ LCHF (low-carb high-fat)<br>☐ 5:2 Diet<br>☐ Church Challenge e.g. Aiga<br><br>☐ Other, please specify ___________________________<br><br>☐ Not applicable: I have not needed to lose weight in the past 12 months<br><br>☐ No. I don't feel I need to lose weight at the moment because:<br><br>____<br><br>☐ No. I have wanted to lose weight but I haven't had been able to do a diet or programme because:<br><br>____<br><br>____

Kia ora. Thank you. You have completed the questionnaire.
All completed questionnaires are to be returned to Dr Marewa Glover: M.Glover@massey.ac.nz

1 What region do you live in?	☐ Northland ☐ Auckland ☐ Manawatu
2 What are your initials?	
3 What is you date of birth?	Month__________________ Year __________________
4 Are you eligible for a Community Services Card?	☐ Yes ☐ No ☐ Don't know
5 How often do you have a drink containing **added** sugar, including but not limited to chocolate milk, milo, soft drink, sports drinks, energy drinks, tea and coffee?<br><br>*Don't include drinks with alcohol. Don't include 'diet' or 'zero' drinks that use artificial sweeteners instead of sugar.*	☐ Don't drink sugar sweetened drinks<br>☐ Once per week<br>☐ 2-3 times per week<br>☐ 4-5 times per week<br>☐ 1 a day<br>☐ 2-3 a day<br>☐ 4 or more a day
6 How much unflavoured water, including sparkling water, do you drink on a usual day?	☐ 0 – 250 mL<br>☐ 500-750 mL<br>☐ 1-1.5L<br>☐ 2 L or more<br>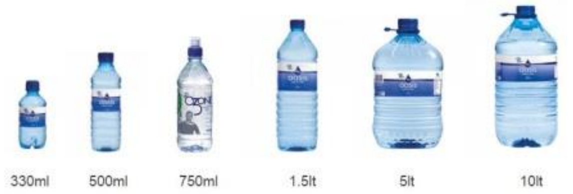
7 During the last 7 days, please circle, on how many days did you eat fruit?<br><br>*Please include all fresh, frozen, canned and stewed fruit. Do not include fruit juice or dried fruit.*	0-----1-----2-----3-----4-----5-----6-----7<br><br>☐ Don't know
8 On average, please circle, how many servings of fruit did you eat each day?<br><br>*A serving is 1 apple or 1 small banana or 2 mandarins or 2 kiwi fruit*	0-----1-----2-----3-----4-----5-----6-----7+
9 During the last 7 days, on how many days did you eat vegetables?	0-----1-----2-----3-----4-----5-----6-----7<br><br>☐ Don't know

10 On average, how many servings of vegetables did you eat each day?<br><br>*A serving is ½ - 1 cup carrots or salad or mixed vegetables*	0-----1-----2-----3-----4-----5-----6-----7+
11 How much margarine, butter or meat fat do you use to season vegetables or put on potatoes, bread or corn?	☐ None<br>☐ 1 level teaspoon ☛ 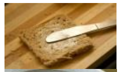<br>☐ 1 heaped teaspoon<br>☐ 1 tablespoon ☛ 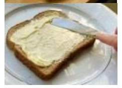<br>☐ 1 heaped tablespoon
12 How often do you choose low or reduced-fat varieties of food instead of the standard variety?	☐ Everyday<br>☐ 2-3 times per week<br>☐ 1-2 times per month<br>☐ Once per month or less
13 How often do you add salt to your food **after** it has been cooked or prepared?	0-----------1--------------2----------------3--------------4<br>Never Rarely Sometimes Often All the time

**During the last 7 days, how many times did you…**	*Circle only one number per line:*
14 …eat fast food meals or snacks? e.g. hot chips, pizza, sausage roll, burger or chicken nuggets	0-----1-----2-----3-----4-----5-----6-----7
15 eat regular snack chips or crackers? e.g. ETA or Bluebird chips	0-----1-----2-----3-----4-----5-----6-----7
16 …eat desserts and other sweets? e.g. cake, puddings, lollies, chocolate or biscuits	0-----1-----2-----3-----4-----5-----6-----7
17 …eat lean meat? e.g. chicken thigh with no skin or unbattered fish or red meat with the fat trimmed	0-----1-----2-----3-----4-----5-----6-----7
18 …eat fresh seafood? e.g. fish, mussels or pipi	0-----1-----2-----3-----4-----5-----6-----7
19 …eat legumes? e.g. butter beans, chickpeas, hummus or lentils	0-----1-----2-----3-----4-----5-----6-----7
20 …have something to eat for breakfast? e.g. porridge or cereal with milk, yogurt with fresh fruit, eggs on toast, a muffin	0-----1-----2-----3-----4-----5-----6-----7
21 What did you have for breakfast this morning? *Write your answer here* ☚	

22 **If you were given a choice, which of the following foods would you choose?** ***(Tick one on each line)***	
1 chicken breast OR 2 chicken drumsticks	
1 lamb shank OR 2 sausages	
4 slices of bread OR 2 rolls	
½ cup pasta or rice OR 1 cup pasta or rice OR 2 cups pasta or rice	
1 kumara OR 2 potatoes OR 1 cup mashed potato	
1 muffin OR 2 slices of cake OR 4 Gingernut biscuits	
23 **Tick the healthier way to cook from each pair:**	
Deep fry OR Stir-fry	
Boiled OR Uncooked / raw	
Steamed OR Boiled	
Shallow fried OR Roasted	
Grilled OR Pan fried	
24 Which **ONE** cooking method do you use the most at home?	
25 Which **ONE** of the above cooking methods do you think is the healthiest of all?	
26 Which **ONE** cooking method do you think is the <u>least</u> healthy of all?	
27 An example of an energy-dense food is:<br>*Tick one*	☐ chocolate ice cream (290 calories per 1 cup)<br>☐ air-popped popcorn (30 calories per 1 cup)<br>☐ sliced fresh strawberries (50 calories per 1 cup)<br>☐ raw carrot sticks (50 calories per 1 cup)

28 Rank these foods, starting from 1 for the lowest number of calories. Calories are a measure of how much energy a food provides.

Write a number from 1 to 10 next to each food in this box,
do not repeat numbers:

____ Pint of beer 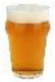

____ Large 150g pack potato chips 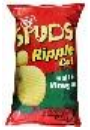

____ An apple 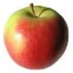

____ An ice cream cone 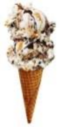

____ A pottle of yoghurt 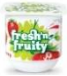

____ A glass of red wine 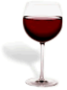

____ Hot chips 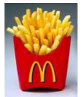

____ A small chocolate bar 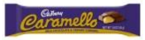

____ A cup of herb tea 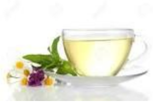

____ Flat white and muffin 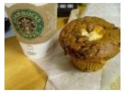

Write a number from 1 to 6 next to each food in this box:
do not repeat numbers:

____ Fish and chips 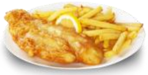

____ Chicken stir-fry with rice 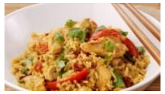

____ Tuna salad 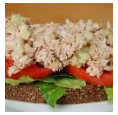

____ A Bic Mac & Fries 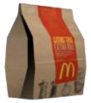

____ Beef stew with potatoes & vegetables 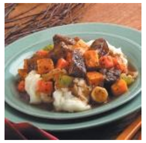

____ A tomato, ham and lettuce sandwich 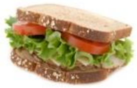

If you could eat one of these ☝ meals now, which one would you choose? *Tick one of the above pictures.*

29 **If you could eat one of the meals in the right hand box now, which one would you choose?** *Tick one of the above pictures.*

**Thinking about the last 7 days:**	*Tick or Circle your answer:*
30 Who in your household usually decides what food and drink is bought?	☐ Me ☐ Me and others jointly<br>☐ Someone else
31 Who in your household usually decides how meals will be cooked?	☐ Me ☐ Me and others jointly<br>☐ Someone else
32 Do you usually dish up your own meals or are your meals plated up for you?	☐ I dish up my own meals<br>☐ Somebody else usually dishes up my plate
33 If you work, do people at work eat lunch together?	0-----------1-------------2---------------3-------------4<br>Never Rarely Sometimes Often All the time<br>☐ Not applicable
34 Food runs out in my/our household.	0-----------1-------------2---------------3-------------4<br>Never Rarely Sometimes Often All the time<br>☐ Don't know
35 I/we make use of special food grants or food banks.	0-----------1-------------2---------------3-------------4<br>Never Rarely Sometimes Often All the time<br>☐ Don't know
36 I feel stressed because of not having enough money for food.	0-----------1-------------2---------------3-------------4<br>Never Rarely Sometimes Often All the time<br>☐ Don't know
37 The variety of food I am/we are able to eat is limited by a lack of money.	0-----------1-------------2---------------3-------------4<br>Never Rarely Sometimes Often All the time<br>☐ Don't know

38 I/we rely on others to provide food and/or money for food for my/our household when I/we don't have enough money.	0-----------1-------------2---------------3-------------4<br>Never Rarely Sometimes Often All the time<br><br>☐ Don't know
39 How often do you have a drink containing alcohol?	☐ Don't drink any alcohol ☛ Go to Q. 41<br>☐ Never or rarely<br>☐ Less than monthly<br>☐ Once a month<br>☐ 2-3 times a month<br>☐ 1-2 times a week<br>☐ 3-4 times a week<br>☐ Once a day<br>☐ 2 times or more a day
40 How many drinks containing alcohol do you have on a typical day when you are drinking?	1-----2-----3-----4-----5-----6-----7-----8------9-----10-----11----12+<br><br>☐ Don't know
41 During the past four weeks, how much of the time have you done less than you would like as a result of any emotional problems, such as feeling depressed or anxious?	0-------------1------------2-------------3-------------4<br>None of the time / A little the time / Some of the time / Most of the time / All of the time<br><br>☐ Don't know

**For questions 42 - 43 “activities” means doing anything using your muscles including activities you do at work, school, home, getting from place to place, for sport, recreation or leisure.**	
42 During the last 7 days, on how many days did you do **moderate** physical activity that made you <u>breathe a little harder</u> than normal?<br><br>*Think **only** about physical activities done for at least 10 minutes at a time.*	0-----1-----2-----3-----4-----5-----6-----7<br><br>☐ Can’t remember
43 What moderate physical activity did you do?	
44 How much time did you typically spend on each of those days doing moderate physical activity?	________ hours (range 0-24) _______ minutes (range 0-60)<br><br>☐ Don’t know
45 During the last 7 days, on how many days did you do **vigorous** physical activity that made you <u>breathe a lot harder</u> than normal (huff and puff)?<br><br>*Think **only** about physical activities done for at least 10mins.*	0-----1-----2-----3-----4-----5-----6-----7<br><br>☐ Can’t remember
46 What vigorous physical activity did you do?	
47 How much time did you typically spend on each of those days doing vigorous physical activity?	________ hours (range 0-24) _______ minutes (range 0-60)<br><br>☐ Don’t know
48 During the last 7 days, usually how many hours sleep each night did you get?	Less than 5---- 5-----6-----7-----8-----9-----10+<br><br>☐ Can’t remember

49 Looking at the pictures below, please circle the **ONE** which you feel is most similar in size to you.

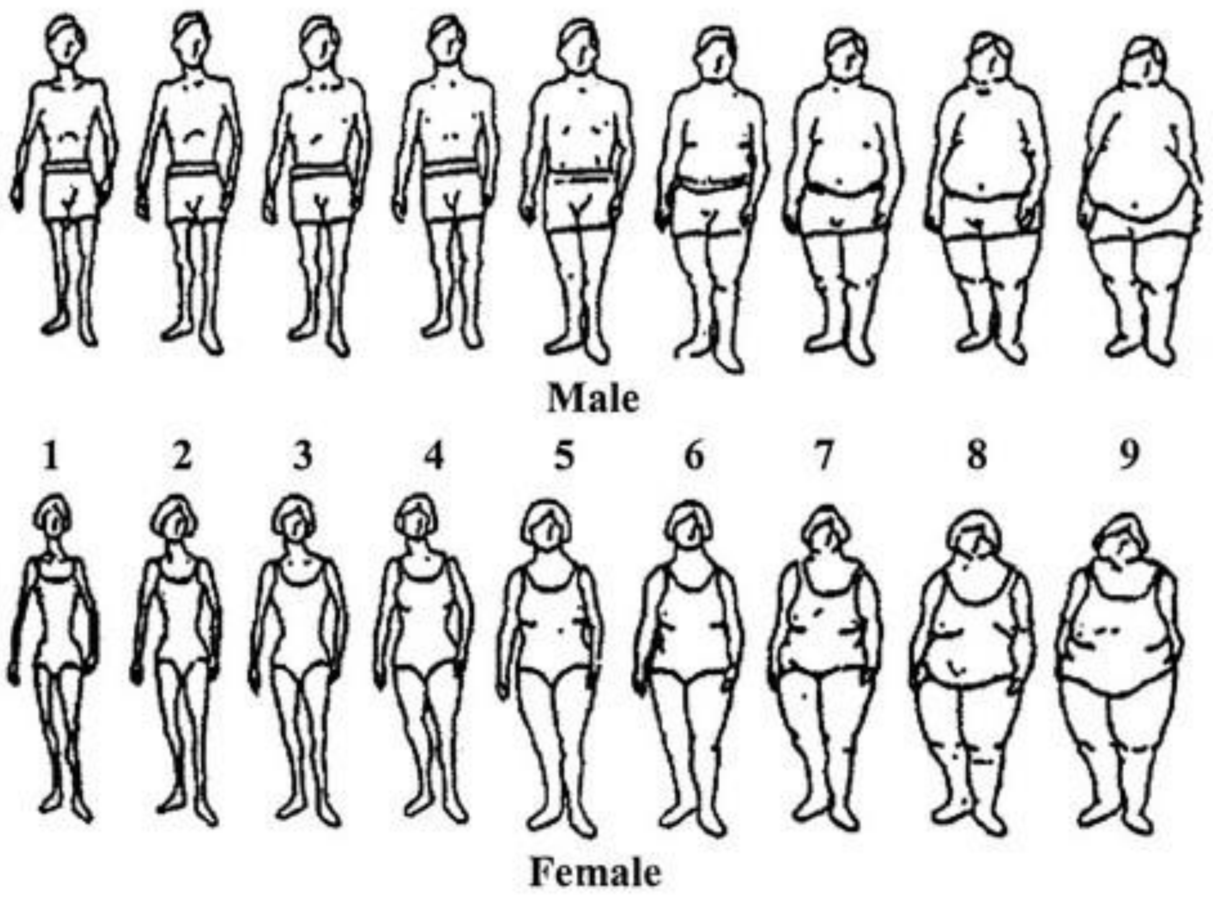

50 Looking at the pictures above, which, if any, of the pictures do you consider are: *(Write the numbers in the space provided)*

____________ underweight

____________ neither under or over weight

____________ a little bit overweight

____________ a lot overweight

____________ seriously overweight

51 Are you **currently** on a weight loss programme or diet such as Weight Watchers, or a diet prescribed by a doctor or dietician?	☐ No<br>☐ Yes. Specify ______________________________
52 In the **past** 12 months, have you used any of the following diets or weight loss programmes to try to lose weight (excluding the one you may currently be following)?	☐ WEHI Programme<br>☐ Weight Watchers<br>☐ Jenny Craig<br>☐ The Paleo Diet<br>☐ Ketogenic Diet<br>☐ Dash Diet<br>☐ LCHF (low-carb high-fat)<br>☐ 5:2 Diet<br>☐ Church Challenge e.g. Aiga<br>☐ Other, please specify ______________________________<br>☐ Not applicable: I have not needed to lose weight in the past 12 months<br>☐ No. I don't feel I need to lose weight at the moment because:<br>______________________________<br>☐ No. I have wanted to lose weight but I haven't had been able to do a diet or programme because:<br>______________________________<br>______________________________

Kia ora. Thank you. You have completed the questionnaire.
All completed questionnaires are to be returned to Dr Marewa Glover: M.Glover@massey.ac.nz

	**WEHI 12 Month Follow Up**	**Today's Date:_________________**
1	What region do you live in?	☐ Northland ☐ Auckland ☐ Manawatu
2	What are your initials?	
3	What is your date of birth	Month ______________ Year _____________
4	Which ethnic group do you belong to?<br>*Tick the box or boxes which apply to you*	☐ Māori<br>☐ Pacific<br>☐ NZ European<br>☐ Other
5	How often do you have a drink containing **added** sugar, including but not limited to chocolate milk, milo, soft drink, sports drinks, energy drinks, tea and coffee?<br>*Don't include drinks with alcohol. Don't include 'diet' or 'zero' drinks that use artificial sweeteners instead of sugar.*	☐ Don't drink sugar sweetened drinks<br>☐ Once per week<br>☐ 2-3 times per week<br>☐ 4-5 times per week<br>☐ 1 a day<br>☐ 2-3 a day<br>☐ 4 or more a day
6	How much unflavoured water, including sparkling water, do you drink on a usual day?	☐ 0 – 250 mL<br>☐ 500-750 mL<br>☐ 1-1.5L<br>☐ 2 L or more<br>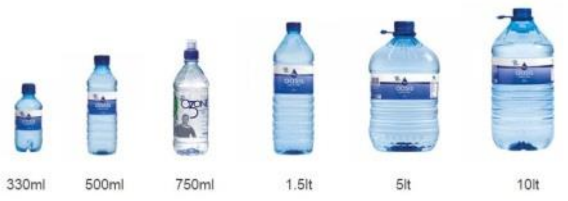
7	During the last 7 days, how many days did you eat fruit?<br>*Please include all fresh, frozen, canned and stewed fruit.*<br>*Do not include fruit juice or dried fruit.*	0-----1-----2-----3-----4-----5-----6-----7<br>☐ Don't know
8	On average, please circle, how many servings of fruit did you eat each day?<br>*A serving is 1 apple or 1 small banana or 2 mandarins or 2 kiwi fruit*	0-----1-----2-----3-----4-----5-----6-----7+

9 During the last 7 days, how many days did you eat vegetables?	0-----1-----2-----3-----4-----5-----6-----7<br>☐ Don’t know
10 On average, how many servings of vegetables did you eat each day?<br>*A serving is ½ - 1 cup carrots or salad or mixed vegetables*	0-----1-----2-----3-----4-----5-----6-----7+
11 How much margarine, butter or meat fat do you use to season vegetables or put on potatoes, bread or corn?	☐ None<br>☐ 1 level teaspoon ☛ 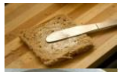<br>☐ 1 heaped teaspoon<br>☐ 1 tablespoon ☛ 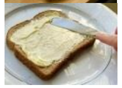<br>☐ 1 heaped tablespoon
12 How often do you choose low or reduced-fat varieties of food instead of the standard variety?	☐ Everyday<br>☐ 2-3 times per week<br>☐ 1-2 times per month<br>☐ Once per month or less
13 How often do you add salt to your food **after** it has been cooked or prepared?	0-----------1-------------2---------------3-------------4<br>Never Rarely Sometimes Often All the time

**During the last 7 days, how many times did you…**	*Circle your answer:*
14 …eat fast food meals or snacks? e.g. hot chips, pizza, sausage roll, burger or chicken nuggets	0-----1-----2-----3-----4-----5-----6-----7
15 eat regular snack chips or crackers? e.g. ETA or Bluebird chips	0-----1-----2-----3-----4-----5-----6-----7
16 …eat desserts and other sweets? e.g. cake, puddings, lollies, chocolate or biscuits	0-----1-----2-----3-----4-----5-----6-----7
17 …eat lean meat? e.g. chicken thigh with no skin or unbattered fish or red meat with the fat trimmed	0-----1-----2-----3-----4-----5-----6-----7
18 …eat fresh seafood? e.g. fish, mussels or pipi	0-----1-----2-----3-----4-----5-----6-----7
19 …eat legumes? e.g. butter beans, chickpeas, hummus or lentils	0-----1-----2-----3-----4-----5-----6-----7
20 …have something to eat for breakfast? e.g. porridge or cereal with milk, yogurt with fresh fruit, eggs on toast, a muffin	0-----1-----2-----3-----4-----5-----6-----7

21 What did you have for breakfast this morning? *Write your answer in the box to the right* ☚	

**22 If you were given a choice, which of the following foods would you choose? *(Circle one on each line)***				
1 chicken breast		OR		2 chicken drumsticks
1 lamb shank		OR		2 sausages
4 slices of bread		OR		2 rolls
½ cup pasta or rice	OR	1 cup pasta or rice	OR	2 cups pasta or rice
1 kumara	OR	2 potatoes	OR	1 cup mashed potato
1 muffin	OR	2 slices of cake	OR	4 Gingernut biscuits

**23 Circle the healthier way to cook from each pair:**		
Deep fry	**OR**	Stir-fry
Boiled	**OR**	Uncooked/raw
Steamed	**OR**	Boiled
Shallow fried	**OR**	Roasted
Grilled	**OR**	Pan fried

24 Which cooking method do you use the most at home?	
25 Which of the above cooking methods do you think is the healthiest of all?	
26 Which cooking method do you think is the <u>least</u> healthy of all?	
27 An example of an energy-dense food is:<br>*Tick one*	☐ chocolate ice cream (290 calories per 1 cup)<br>☐ air-popped popcorn (30 calories per 1 cup)<br>☐ sliced fresh strawberries (50 calories per 1 cup)<br>☐ raw carrot sticks (50 calories per 1 cup)

**Thinking about the last 7 days:**	*Tick or Circle your answer:*
28 Who in your household *usually* decides what food and drink is bought?	☐ Me ☐ Me and others jointly<br>☐ Someone else
29 Who in your household *usually* decides how meals will be cooked?	☐ Me ☐ Me and others jointly<br>☐ Someone else
30 Do you *usually* dish up your own meals or are your meals plated up for you?	☐ I dish up my own meals<br>☐ Somebody else usually dishes up my plate
31 If you work, do people at work eat lunch together?	0----------1-------------2---------------3-------------4<br>Never Rarely Sometimes Often All the time<br><br>☐ Not applicable
32 Food runs out in my/our household.	0----------1-------------2---------------3-------------4<br>Never Rarely Sometimes Often All the time<br><br>☐ Don't know
33 I/we make use of special food grants or food banks.	0----------1-------------2---------------3-------------4<br>Never Rarely Sometimes Often All the time<br><br>☐ Don't know
34 I feel stressed because of not having enough money for food.	0----------1-------------2---------------3-------------4<br>Never Rarely Sometimes Often All the time<br><br>☐ Don't know
35 The variety of food I am/we are able to eat is limited by a lack of money.	0----------1-------------2---------------3-------------4<br>Never Rarely Sometimes Often All the time<br><br>☐ Don't know

36 I/we rely on others to provide food and/or money for food for my/our household when I/we don't have enough money.	0-----------1--------------2----------------3--------------4<br>Never Rarely Sometimes Often All the time<br><br>☐ Don't know
37 How often do you have a drink containing alcohol?	☐ Don't drink any alcohol ☛ Go to Q. 39<br>☐ Never or rarely<br>☐ Less than monthly<br>☐ Once a month<br>☐ 2-3 times a month<br>☐ 1-2 times a week<br>☐ 3-4 times a week<br>☐ Once a day<br>☐ 2 times or more a day
38 How many drinks containing alcohol do you have on a typical day when you are drinking?	1-----2-----3-----4-----5-----6-----7-----8------9-----10-----11----12+<br><br>☐ Don't know
39 During the past four weeks, how much of the time have you done less than you would like as a result of any emotional problems, such as feeling depressed or anxious?	0--------------1-------------2--------------3--------------4<br>None of the time / A little the time / Some of the time / Most of the time / All of the time<br><br>☐ Don't know

For questions 40 - 46 **“activities” means doing anything using your muscles including activities you do at work, school, home, getting from place to place, for sport, recreation or leisure.**	
40 During the last 7 days, on how many days did you do **moderate** physical activity that made you breathe a little harder than normal?<br>*Think **only** about physical activities done for at least 10 minutes at a time.*	0-----1-----2-----3-----4-----5-----6-----7<br>☐ Can’t remember
41 What moderate physical activity did you do?	
42 How much time did you typically spend on each of those days doing moderate physical activity?	________ hours (range 0-24) _______ minutes (range 0-60)<br>☐ Don’t know
43 During the last 7 days, on how many days did you do **vigorous** physical activity that made you breathe a lot harder than normal (huff and puff)?<br>*Think **only** about physical activities done for at least 10mins.*	0-----1-----2-----3-----4-----5-----6-----7<br>☐ Can’t remember
44 What vigorous physical activity did you do?	
45 How much time did you typically spend on each of those days doing vigorous physical activity?	________ hours (range 0-24) _______ minutes (range 0-60)<br>☐ Don’t know
46 During the last 7 days, usually how many hours sleep each night did you get?	Less than 5---- 5-----6-----7-----8-----9-----10+<br>☐ Can’t remember

47 In the **past 6 months**, since the end of the WEHI competition, have you used any of the following diets or weight loss programmes to try to lose weight?	☐ Weight Watchers<br>☐ Jenny Craig<br>☐ The Paleo Diet<br>☐ Ketogenic Diet<br>☐ LCHF (low-carb high-fat)<br>☐ 5:2 Diet<br>☐ Church Challenge e.g. Aiga<br>☐ Other, please specify ______<br><br>☐ Not applicable: I have not needed to lose weight in the past 6 months<br><br>☐ No. I don't feel I need to lose weight at the moment because:<br>______<br>______<br>______<br><br>☐ No. I have wanted to lose weight but I haven't had been able to do a diet or programme because:<br>______<br>______<br>______

48 Many of the participants were not able to do the WEHI challenge for the whole 6 months. If you stopped doing the daily challenges during the WEHI competition, which of these reasons apply to you?<br>*Tick **all** that apply.*	☐ Injury / illness ☐ Doctor advised me to stop<br>☐ Couldn't find time to do ☐ I lost motivation<br>☐ Other responsibilities came first<br>☐ My team couldn't get it together<br>☐ My family didn't support me enough<br>☐ I did the challenges but didn't lose weight<br>☐ It was too hard to catch up with the winning team<br>☐ Other, please specify ______________________<br>☐ Not applicable: I did the WEHI challenges over the whole 6 months of the competition.
49 Which of these choices would have made WEHI better:<br>*Tick **one only***	☐ The total prize money is won by the leading team?<br>**OR**<br>☐ The total prize money is divided among the teams proportionate to their team points? (The team with the most points gets the biggest share, while team with least points gets the least less).
50 Which of these choices would have made WEHI better:<br>*Tick **one only***	☐ The prize money goes to the team's charity?<br>**OR**<br>☐ The prize money goes to the team for them to spend how they like?

Kia ora. Thank you. You have completed the WEHI study.
